# Protecting against genetic discrimination requires more than a law

**DOI:** 10.1016/j.lanwpc.2026.101975

**Published:** 2026-08-31

**Authors:** The Lancet Regional Health – Western Pacific

On Oct 8, 2026, Australia will ban life insurance companies nationwide from collecting genetic testing results when reviewing applications. The bill has been widely welcomed by stakeholders, including the Australian Medical Association, the Council of Australian Life Insurers, and the Australian Human Rights Commission. For the many who have hesitated to take a genetic test or participate in genomic research for fear of being refused insurance because of the results, the new law is a significant win against genetic discrimination.

Genetic testing used to be offered mainly to symptomatic patients for diagnostic purposes. However, as the costs of genetic testing have fallen, its use has become increasingly popular among healthy individuals and those with a family history who wish to proactively understand their own underlying risk of diseases for prevention. Common direct-to-consumer tests can identify carriers of recessive conditions such as cystic fibrosis before starting a family, *BRCA1/2* gene mutations that increase the risk of developing breast and ovarian cancers, and provide pharmacogenomic information that guides the choice of medication for optimal absorption and metabolism. As these results are closely tied to health conditions, many people fear the information could backfire in the form of genetic discrimination (ie, the unfair treatment of individuals because of their genetic make-up), such as the risk of a genetic disorder. A population-based study reported that when told how genetic testing might affect insurance underwriting, people were 50% less likely to proceed with testing. On the contrary, a Japanese survey revealed that insurers were worried about actuarial fairness and “adverse selection”, by which people with hidden health issues exploit information gaps to withhold their details when taking out insurance, which eventually drives up premiums for all policy holders.

From a medical ethics perspective, the answer to this dilemma is straightforward: no one should be forced to choose between prioritising their health and financial security. This is particularly unfair when the genetic information used can be less predictive than we assume. For example, the reliability of a genomic risk-prediction tool in an Asian population increases when it has been externally validated in the region, as gene variants might be Asian-specific but most existing genetic data have been derived from populations of European ancestry. Although monogenic disorders strongly determine whether a disease develops, most allelic variants only marginally contribute to overall risk. Even with the development of polygenic risk scores, they should be used together with traditional risk factors to give accurate risk projections, as managing lifestyle factors is shown to attenuate the associations. Genetic testing is therefore meant to direct individuals at higher risk towards more intensive prevention programmes for risk mitigation, rather than assigning them a permanent label.

Banning genetic discrimination in the Western Pacific is not simple, partly because insurance systems are heterogeneous across the region. A community-rated system charges everyone the same base premium for the same product, whereas some jurisdictions, such as Hong Kong, typically adopt a risk-rated approach that adjusts premiums according to granular, individual-level risk factors. This raises a question: does the practice of risk adjustment based on health-related information contribute to discrimination? Governments in these jurisdictions should therefore clarify in law how genetic data are classified to distinguish between general health disclosure and sensitive personal details. In Singapore, a partial ban has been implemented to exempt Huntington's disease and *BRCA1/2* findings, which still discourages testing as no one knows beforehand whether they would fall into an exempted category.

Genetic discrimination also appears in settings other than insurance. In 2009, 31 job applicants in China were refused employment for carrying a thalassaemia gene regardless of physical fitness. The Government later introduced measures to ban the practice, but rather focused on the context of insurance. To date, legal protection across the region remains patchy, as only South Korea and Japan have passed broad laws against genetic discrimination with criminal sanctions. Even where legislation exists, the bias remains in many social activities not covered by law. In Japan, 36% of people were concerned about being judged based on their genetics in marriage when choosing partners. This suggests that bias also blends into cultural and personal decisions, and that the public is also a key stakeholder on this topic.

Much remains to be done beyond legislation given the diverse systems and social settings in the region. Public understanding also matters, as genetics are increasingly communicated at the personal level. In aspects where legislation cannot reach, researchers and test providers bear an ethical responsibility to help participants interpret their risk profile carefully, and individuals should be judged on their actual abilities instead of being penalised for a test result that only estimates probability. Preventing genetic discrimination requires not only passing a single law, but also a society literate enough to read the science without fear.

